# Histologic evaluation of activation of acute inflammatory response in a mouse model following ultrasound-mediated blood-brain barrier using different acoustic pressures and microbubble doses

**DOI:** 10.7150/ntno.49898

**Published:** 2020-07-14

**Authors:** Aurea Pascal, Ningrui Li, Kendra J. Lechtenberg, Jarrett Rosenberg, Raag D. Airan, Michelle L. James, Donna M. Bouley, Kim Butts Pauly

**Affiliations:** 1 Department of Radiology, Stanford University, Stanford, California 94305, USA.; 2 Department of Electrical Engineering, Stanford University, Stanford, California 94305, USA.; 3 Department of Neurology and Neurological Sciences, Stanford University, Stanford, California 94305, USA.; 4 Department of Comparative Medicine, Stanford University, Stanford, California 94305, USA.

**Keywords:** blood-brain barrier, focused ultrasound, microbubbles, acute neuroinflammation, immunohistochemistry

## Abstract

**Rationale:** Localized blood-brain barrier (BBB) opening can be achieved with minimal to no tissue damage by applying pulsed focused ultrasound alongside a low microbubble (MB) dose. However, relatively little is known regarding how varying treatment parameters affect the degree of neuroinflammation following BBB opening. The goal of this study was to evaluate the activation of an inflammatory response following BBB opening as a function of applied acoustic pressure using two different microbubble doses.

**Methods:** Mice were treated with 650 kHz ultrasound using varying acoustic peak negative pressures (PNPs) using two different MB doses, and activation of an inflammatory response, in terms of microglial and astrocyte activation, was assessed one hour following BBB opening using immunohistochemical staining. Harmonic and subharmonic acoustic emissions (AEs) were monitored for all treatments with a passive cavitation detector, and contrast-enhanced magnetic resonance imaging (CE-MRI) was performed following BBB opening to quantify the degree of opening. Hematoxylin and eosin-stained slides were assessed for the presence of microhemorrhage and edema.

**Results:** For each MB dose, BBB opening was achieved with minimal activation of microglia and astrocytes using a PNP of 0.15 MPa. Higher PNPs were associated with increased activation, with greater increases associated with the use of the higher MB dose. Additionally, glial activation was still observed in the absence of histopathological findings. We found that CE-MRI was most strongly correlated with the degree of activation. While acoustic emissions were not predictive of microglial or astrocyte activation, subharmonic AEs were strongly associated with marked and severe histopathological findings.

**Conclusions:** Our study demonstrated that there were mild histologic changes and activation of the acute inflammatory response using PNPs ranging from 0.15 MPa to 0.20 MPa, independent of MB dose. However, when higher PNPs of 0.25 MPa or above were applied, the same applied PNP resulted in more severe and widespread histological findings and activation of the acute inflammatory response when using the higher MB dose. The potential activation of the inflammatory response following ultrasound-mediated BBB opening should be considered when treating patients to maximize therapeutic benefit.

## Introduction

Ultrasound-mediated blood-brain barrier (BBB) opening temporarily increases local vascular permeability, allowing delivery of therapeutic agents previously unable to penetrate the brain parenchyma for the potential treatment of neurological diseases 1-5. Localized BBB opening can be achieved by targeting specific brain regions with low intensity pulsed focused ultrasound (pFUS) while simultaneously administering systemic microbubbles (MBs). This technique provides an alternative to more invasive treatment methods, and clinical trials are currently underway to investigate its safety and efficacy in patients with Alzheimer's disease 6 or glioma 7,8.

Several studies have assessed the safety of BBB opening in a range of animal models. Histological analyses have focused on the presence or absence of hemorrhage and edema a few hours or days post single 9-11 or multiple treatments 12-15. Results have demonstrated that BBB opening can be achieved with little to no hemorrhage or edema assuming optimal acoustic pressures are applied alongside a sufficiently low MB dose, such as those used for clinical imaging (10 μL per kg).

Ultrasound-mediated BBB opening has been shown to result in varying degrees of neuroinflammation, despite minimal tissue damage. 16-20. Using a rat model, Kovacs et al. demonstrated that there was upregulation of pro- and anti-inflammatory cytokines and neurotrophic factors following BBB opening using a constant acoustic pressure and a relatively high MB dose (~500 μL *Optison* per kg). This treatment also resulted in microglial and astrocyte activation as well as changes in gene expression in the NFκB signaling pathway. Conversely, McMahon et al. observed that the expression of genes in the NFκB signaling pathway was substantially reduced when using a low MB dose while dynamically controlling the applied acoustic pressure using a passive cavitation detection-based feedback system 17.

However, these studies did not address the significance of the interaction between MB dose and applied acoustic pressure in causing neuroinflammation following pFUS and MB administration. Therefore, the purpose of this study was to evaluate activation of an acute inflammatory response, assessed through microglial and astrocyte activation, over a range of applied acoustic pressures with two different MB doses at one-hour post BBB opening. To that end, we treated mice using varying peak negative acoustic pressures (PNPs) at two different MB dosages: 10 μL/kg and 250 μL/kg. We evaluated microglial and astrocyte activation one-hour post treatment using histological examination of treated mouse brain. At both MB dosages, the selected range of applied PNPs yielded a wide distribution of treatment outcomes: low applied PNPs did not result in BBB opening, whereas high applied PNPs resulted in BBB opening accompanied by severe perivascular microhemorrhage and edema.

Even when using the same treatment parameters, the* in situ* acoustic pressures can differ greatly across human patients due to high inter-subject variability of skull geometry and composition 21,22. Therefore, a secondary aim of our study was to assess whether the degree of glial activation or histopathology were correlated with outcome metrics that do not require knowledge of the *in situ* acoustic pressure, such as relative signal enhancement on contrast-enhanced magnetic resonance imaging (CE-MRI) or acoustic emissions (AEs) from passive cavitation detection.

## Materials and Methods

### Animals

All *in vivo* mouse experiments were approved by the Stanford Institutional Animal Care and Use Committee. All mice were treated in accordance with the Guide for the Care and Use of Laboratory Animals in an AAALAC accredited facility.

Sixty female CD1 mice (7-9 weeks old, 20-40 g weight, Charles River Laboratories, Boston, MA) acclimated to the facility for 4-6 days prior to experimental procedures were housed on a 12-hour light/dark cycle in groups of 2-5 mice per cage, with *ad libitum* food and water. The room temperature was maintained at 23°C ± 1°C with a relative humidity of 55% ± 5%.

### Experimental design

Animals were randomly assigned to twelve treatment groups, with five individuals per group. Four treatment groups received an intravenous (IV) bolus injection of MBs (*Definity*, Lantheus Medical Imaging Inc., N. Billerica, MA) at 10 μL per kg (the clinical imaging dose) alongside sonications with PNPs of either 0.15 MPa, 0.25 MPa, 0.35 MPa, or 0.45 MPa (the upper end of the pressure average was increased to 0.45 MPa in order to include the same range of histopathological lesions that were seen at PNP of 0.35 MPa and higher dose of MB). Six treatment groups received a higher dose of microbubbles (250 μL per kg, 25× the dose recommended for clinical imaging) and sonications with PNPs of either 0.10 MPa, 0.15 MPa, 0.20 MPa, 0.25 MPa, 0.30 MPa, or 0.35 MPa. Negative control animals received either pFUS-only, sonicated at a PNP of 0.35 MPa with IV saline instead of MBs, or imaged but with no pFUS or MBs. At a sonication frequency of 650 kHz, the insertion loss due to the mouse skull was approximately 7% 23. For simplicity, all stated PNP values are non-derated and refer to the PNP at the focus measured using a fiber-optic hydrophone (Precision Acoustics, Dorset, UK) while sonicating in free water.

### BBB opening protocol

Mice were anesthetized using 3% isoflurane (carried on 1.5% oxygen). Fur on their heads was removed using an electric razor and depilatory cream (*Nair*, Church & Dwight, USA) and a 27-gauge butterfly catheter with tubing (SAI Infusion Technologies) was inserted in the tail vein for administration of MB or saline solution as well as gadolinium contrast agent. Mice were then placed into a stereotactic head frame and maintained on 1.5% to 2% isoflurane (carried on 1.5% medical air) for approximately fifteen minutes while a mechanical positioning system (Image Guided Therapy, Bordeaux, France) was used to place the transducer to target the left cerebral hemisphere. The target coordinates were approximately 8 mm caudal to the eyes and 3 mm left of the sagittal suture. Respiratory rate, mucous membrane color, and pedal reflex were monitored every 10 minutes, and the anesthesia level was adjusted to maintain a respiratory rate between 20 and 30 breaths/minute.

A 650 kHz six-element annular array (focal depth, radius of curvature, transducer diameter = 30 mm) ultrasound transducer (IMASONIC, Voray-sur-l'Ognon, France) was used for all sonications (pulse repetition frequency = 1 Hz, burst length = 10 ms, total duration = 120 s). Five pulses were applied prior to administration of MB or saline solution, and the cavitation spectra from these pulses served as a baseline for computing AEs. Immediately prior to injection, MB vials were activated at room temperature using the *Vialmix* agent activator (Lantheus Medical Imaging Inc., N. Billerica, MA) and diluted with saline (0.9% sodium chloride, Hospira Farris Lab, TX, USA) at ratios of 1:500 v/v for a dose of 10 µL MB/kg or 1:20 v/v for a dose of 250 µL MB/kg. The tail vein catheter tubing was cut to 3 cm long and either saline or diluted MB were administered via the tail-vein as a slow bolus injection (5 to 10 sec) simultaneously with application of pFUS. In all mouse groups, the total fluid volume given was 5 µL diluted solution per g.

### MR imaging

Immediately following pFUS, mice were imaged at 3T (MRS3000, MR Solutions, Guildford, Surrey, UK). T_1_-weighted fast spin-echo MR images (axial and coronal views, repetition time: 720 ms, echo time: 11 ms, echo train length: 4, field of view: 25 mm by 25 mm, acquisition matrix: 256 × 252, slice thickness: 0.6 mm, flip angle: 90°, number of averages: six) were acquired before and after administration of gadolinium contrast agent (*MultiHance*, BRACCO Diagnostics Inc, Monroe Township, NJ). Contrast agent was administered intravenously at a dose of 0.1 µL per g.

The degree of BBB opening was quantified using the contrast-enhanced T_1_-weighted images. Two brain regions (selected from either the cortex or thalamus) in the sonicated hemisphere were chosen for each animal where local peaks in signal intensity were observed. 0.5 mm by 0.5 mm regions were centered on these signal intensity peaks, and mean signal intensities were computed within each region in the sonicated hemisphere. Mean signal intensities were also computed for the contralateral regions in the untreated hemisphere. The relative signal enhancement was calculated as a ratio between the mean signal intensity in the region within the sonicated hemisphere and the mean signal intensity in the region within the untreated hemisphere.

### Subharmonic and harmonic acoustic emissions

AEs were measured for each sonication using a single-element PCD (1.95 MHz center frequency, 51% fractional bandwidth) embedded in the center of the element array. Raw voltage traces from the PCD were recorded with an oscilloscope at a sampling frequency of 6.58 MHz (Pico Technology, Cambridgeshire, UK) and processed using MATLAB (Mathworks, Inc., Natick, MA). The magnitude spectrum of each received acoustic signal was computed using the fast Fourier transform algorithm.

Subharmonic AEs were computed by averaging the magnitude spectrum of the received acoustic signal over a wider band (325 kHz ± 2 kHz) while excluding a narrower band (325 kHz ± 100 Hz). A sonication was considered to contain subharmonic AEs if this average was at least 7 dB higher than the noise floor, which was defined as the median magnitude value over the entire spectrum. Harmonic AEs were computed similarly by averaging the magnitude spectrum over a narrow band (1.3 MHz ± 300 Hz). The baseline level of harmonic AEs was computed for each animal by averaging harmonic AEs from five sonications made prior to MB or saline administration. The increase in harmonic AEs relative to this baseline was computed for all sonications post administration of MB or saline solution.

### Mouse perfusion and brain extraction

Following MR imaging, each anesthetized mouse was transported to a fume hood and intracardiac whole-body perfusion with 4% paraformaldehyde (PFA) (Santa Cruz Biotechnology, Dallas, TX) using a *MASTERFLEX* pump drive (Cole-Parmer Instrument Company LLC, Vernon Hills, IL) was performed. The perfusion rate was 3 mL/min with PBS for two minutes (Gibco Fisher Scientific, Hampton, NH), followed by fifteen minutes with PFA. Each fixed brain was extracted and post-fixed in 4% PFA for 48 hours at 4 °C, then placed in 70% alcohol (Thermo Scientific, Waltham, MA) and maintained at 4 °C. At the time of harvest, each brain was inspected for any grossly visible lesions.

### Histopathology

Whole brains were placed in a mold and coronal cuts made using Thompson blades (Thomas Scientific, Swedesboro, NJ). When present, the locations of hemorrhage observed on the surface of the brain as well as enhancing brain regions on CE-MRI were used to ensure that slices were located at the center of the treated brain region (Figure S1). When there was no observable tissue damage or contrast enhancement on CE-MRI, target planning information from the pFUS mechanical positioning system was used as guidance to ensure that the slice was centered at the treated area.

Three coronal cuts were made, yielding two slices of 3 mm thick tissue. These slices were routinely processed for paraffin embedding, serially cut on a microtome at 5 µm thickness, and mounted on *SuperFrost Plus* slides. A subset of slides were stained with hematoxylin and eosin (H&E), and adjacent slides were left unstained for immunohistochemistry. Stained slides were scanned on a brightfield digital scanner (Nanozoomer2.0RS, Hamamatsu Photonics, Japan) at 20X magnification.

One H&E-stained slide per animal was reviewed by a board-certified veterinary pathologist (DMB) who was blinded to treatment groups. Sections were evaluated for evidence of microhemorrhages and edema using a semiquantitative scoring system (Table 1). When lesions were present, they consisted of varying degrees of capillary disruption, red blood cell extravasation (microhemorrhages), and small to regionally extensive edema of the neuropil (extracellular brain tissue). The untreated hemisphere served as a negative control for all brains.

### Immunohistochemistry

To better visualize morphologic changes resulting from activation of microglia and astrocytes, immunohistochemical (IHC) staining was performed according to the manufacturer's instructions on paraffin-embedded coronal brain slices 24,25. Briefly, sections were deparaffinized using xylene and decreasing graded concentrations of alcohol (100%-50%), then rinsed with Tris-buffered saline (TBS) (Biocare Medical, Pacheco, CA). Heat-induced antigen retrieval was performed in a sodium citrate buffer at a pH of 6 (Rodent Decloaker 1X, Biocare Medical, Pacheco, CA). Slides were cooled, rinsed, and incubated with *Peroxidazed 1* (Biocare Medical, Pacheco, CA) and a protein blocker (*Background Punisher*, Biocare Medical, Pacheco, CA) for ten minutes to reduce non-specific staining.

Slides were incubated overnight at 4°C with primary antibodies directed toward ionized calcium-binding adapter molecule 1 (Iba1), which are expressed in microglia, or glial fibrillary acidic protein (GFAP), which are expressed in astrocytes. The presence of infiltrating macrophages has been reported following BBB opening 16. To distinguish microglia from infiltrating macrophages, antibodies directed toward transmembrane protein 119 (TMEM119) were also used, as TMEM119 expression has been shown to be more specific to microglia 26. Anti-Iba1 rabbit monoclonal antibodies (abcam, Cambridge, MA) were used at a concentration of 1.27 mg/mL (1:1000 v/v dilution), while anti-TMEM119 rabbit monoclonal antibodies (abcam, Cambridge, MA) were used at a concentration of 0.153 mg/mL (1:100 v/v dilution) and anti-GFAP mouse monoclonal antibodies (abcam, Cambridge, MA) were used at a concentration of 0.05 mg/mL (1:100 v/v dilution). All antibodies were diluted with 1% goat serum in TBS (Biocare Medical, Pacheco, CA). Negative controls for all IHC utilized IgG1/kappa universal negative control serum (Biocare Medical, Pacheco, CA).

The following day, slides were rinsed, and secondary rabbit-on-rodent or mouse-on-mouse detection systems (Rabbit on Rodent HRP-Polymer or MACH 4 Universal AP Polymer Kit, Biocare Medical, Pacheco, CA) were applied to the slides and incubated for one to four hours at room temperature. Sections were then washed and stained with DAB horseradish peroxidase (HRP) substrate (Vector Laboratories, Burlingame, CA) and counterstained with hematoxylin (CAT Hematoxylin, Biocare Medical, Pacheco, CA). Finally, slides were digitally scanned as previously described.

**Figure 1:**
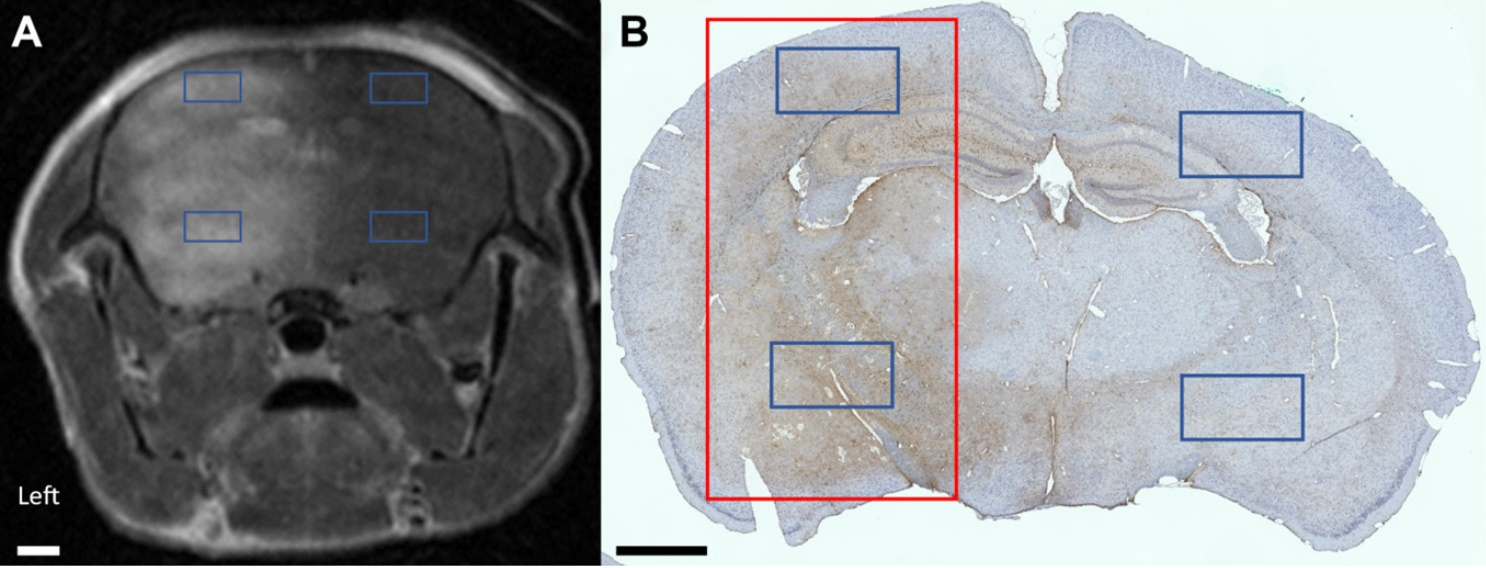
(A) Coronal CE-MRI slice and (B) the corresponding IHC DAB-stained paraffin-embedded slide. The coronal CE-MRI slice was flipped from radiological convention for clearer comparison to the IHC-stained slide. The full-width half-maximum of the focal spot, representing the treated brain region, is denoted in red. Signal intensity on CE-MRI as well as the number of activated microglia and astrocytes were quantified in four 1.32 mm × 0.72 mm ROIs (denoted in blue): two in the sonicated hemisphere and two in the untreated hemisphere. The scale bar in both images represents 1 mm.

To evaluate microglial and astrocyte activation within the treated region, 1.32 mm by 0.72 mm regions of interest (ROIs) were first located based on contrast-enhancing regions of coronal CE-MRI slices. Next, the corresponding ROIs were identified in the IHC-stained brain slice (Figure 1). Within each slice, two brain regions were assessed: one in the cortex and one in the thalamus. The contralateral regions in the untreated hemisphere were also evaluated, for a total of four ROIs.

**Figure 2:**
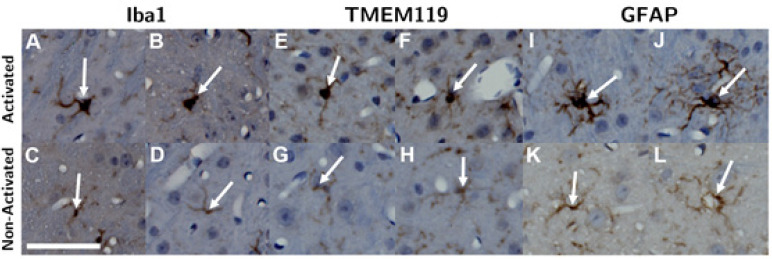
Microglia were identified by Iba1 (A - D) and TMEM119 (E - H) staining, and astrocytes were identified by GFAP (I - L) staining. Next, activated microglia and astrocytes (top row) were differentiated from non-activated ones (bottom row) based on hypertrophic changes observed in their cell body and thickening of their processes. The length of the scalebar is 50 µm.

Within each ROI, microglia and astrocytes were first identified by their staining intensity. Activated cells were then differentiated from non-activated ones based on morphology (Figure 2). Microglia (both activated and non-activated) were first located based on staining intensity of anti-Iba1 antibodies. Next, activated microglia were identified as activated by hypertrophic changes in their morphology, which included enlarged and more rounded cell bodies as well as thicker and “bushier” main processes 27-32. This process was repeated in another adjacent slice stained with anti-TMEM119 antibodies. Similarly, astrocytes were first identified based on staining intensity with anti-GFAP antibodies, then they were classified as activated or non-activated based on hypertrophic morphological changes and increased thickness of processes 33-35 (Figure 2).

### Statistical analysis

As signal enhancement was expected to saturate at low applied PNPs and high applied PNPs, the Gompertz model was chosen to estimate the relative signal enhancement on CE-MRI as a function of applied PNP, and optimal model parameters were computed using non-linear least-squares regression with the Levenberg-Marquardt method. Correlations between activated cell counts and PNP, relative CE-MRI signal enhancement, and harmonic AEs were assessed using least-squares linear regression. Regression analyses were performed using the *scipy* (version 1.2.1), the *statsmodels* (version 0.9.0), and *lmfit* (version 1.0.0) modules in Python.

Next, relationships between the applied PNP and histopathological grading were assessed by Kendall's tau correlation and tested by Fisher's exact test. The average difference in the number of activated microglia and astrocytes between region pairs in sonicated vs. untreated hemispheres were computed and assessed for statistical significance using the Wilcoxon paired-sample signed-rank test with the Pratt modification. Finally, relationships between histopathological grading and activated cell counts were tested by the Jonckheere-Terpstra non-parametric test of trend. Non-parametric tests were chosen as assumptions regarding normality and equal variances could not be reliably confirmed due to the small number of animals in each treatment group. These analyses were performed using Stata Release 15.1 (StataCorp LP, College Station, TX) and the *clusrank* package in R 36. A significance level of 0.05 was used for all analyses.

## Results

### Wide range of treatment outcomes achieved by varying PNPs for both MB doses

**Figure 3:**
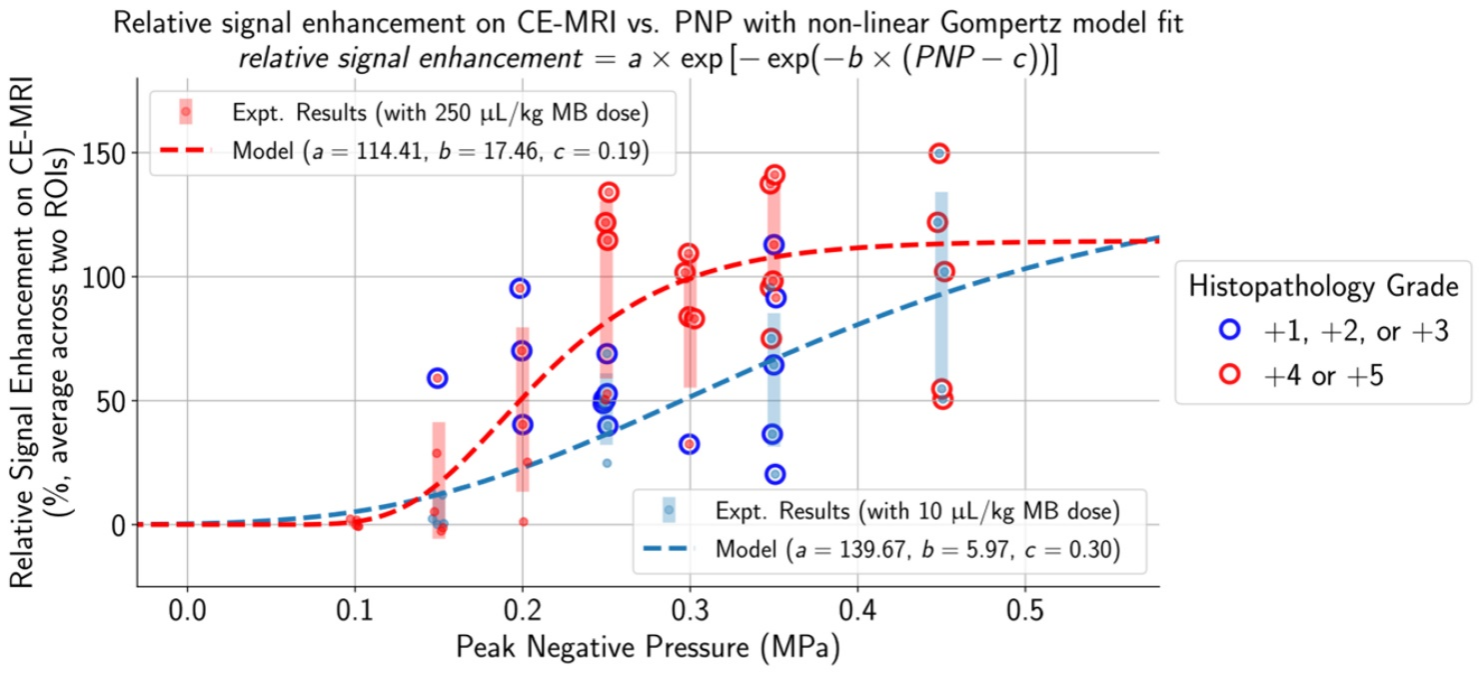
The selection of applied PNPs for both MB doses resulted in a comprehensive range of treatment outcomes. At low applied PNPs, contrast enhancement was not seen on CE-MRI and there were no histopathological findings in the targeted brain region. In contrast, there were multiple affected regions with edema and microhemorrhages and high relative contrast enhancement on CE-MRI when applying high PNPs. Increased histopathological findings (blue outline: histopathology grades of +1/+2/+3 and red outline: histopathology grades of +4/+5) and higher relative signal enhancement on CE-MRI were observed when using the higher MB dose at the same applied PNP. The non-linear Gompertz regression model (dashed lines, mean absolute percent error of 6.79% for the 10 µL/kg model and 0.83% for the 250 µL/kg model, p < 0.05 for all model parameters) estimated that relative signal enhancement on CE-MRI increases with higher applied PNP for animals treated with both MB doses. Midlines are situated at the mean for each group, and error bars represent standard deviations.

Applied PNPs were chosen to establish a range of treatment outcomes for both low and high MB doses. As observed on histopathology (Figure 3, Table S1), lesion frequency and severity were significantly associated with increasing PNP, ranging from no lesions at low applied PNPs to multiple areas exhibiting primarily perivascular microhemorrhages and edema at higher applied PNPs.

With a MB dose of 250 μL/kg and a low applied PNP of 0.10 MPa, there were no lesions observed on histopathology, the BBB remained intact, and relative signal enhancement was negligible. As the applied PNP increased to 0.15 MPa, BBB opening was observed on CE-MRI in two out of five mice when using a MB dose of 10 μL/kg and three out of five mice when using a MB dose of 250 μL/kg (Table S1). At the higher end of applied PNPs, the degree of BBB disruption was substantial and associated with severe microhemorrhage and edema. This began at 0.45 MPa when a MB dose of 10 μL/kg was administered and at 0.25 MPa when using the higher 250 μL/kg dose. Microhemorrhage, edema, or BBB opening were not observed in any control mice or mice given pFUS only (0.35 MPa PNP), and pathological changes were not present in the untreated hemispheres of any animals.

### Higher PNP associated with activation of microglia and astrocytes regardless of MB dose

**Figure 4:**
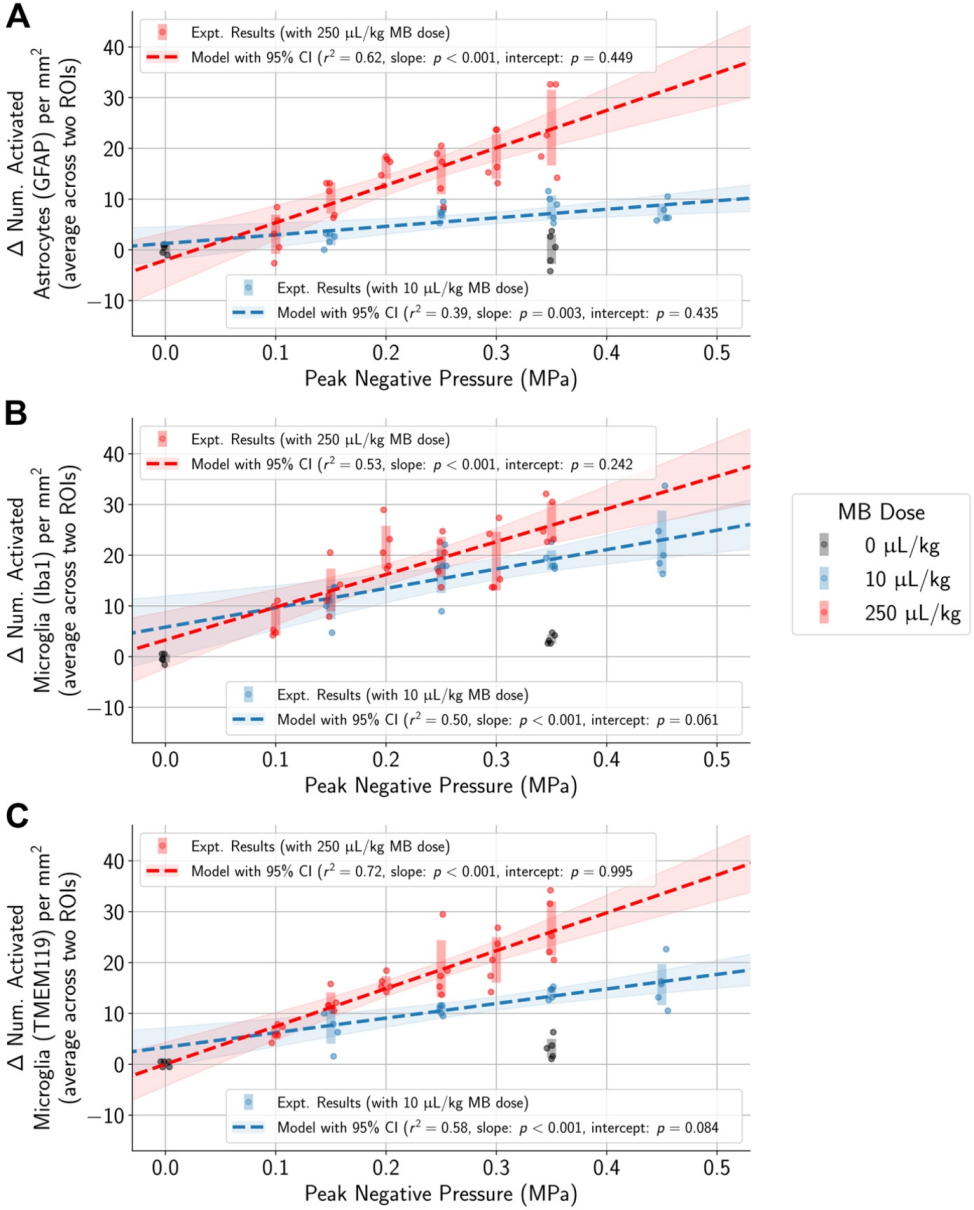
In animals administered MB, differences in the number of activated microglia and astrocytes per mm^2^ between the sonicated and untreated hemispheres were correlated with applied PNP (highlighted regions represent 95% confidence intervals for the mean, and error bars represent standard deviations for each PNP group). Statistically significant differences between animals given either the 10 µL/kg or 250 µL/kg MB dose were observed only at higher applied PNPs, but not with PNPs of 0.15 MPa or below.

To control for variability across animals and different regions of the brain, the difference in the numbers of activated microglia and astrocytes between each pair of ROIs in the sonicated and untreated hemispheres was computed. Differences in the numbers of activated cells increased with higher applied PNPs (Figure 4, Table S2). Overlaps in 95% confidence intervals suggest that at low applied PNPs of 0.15 MPa or below, differences in activated cells between sonicated vs. untreated hemispheres were not significantly different when using the lower MB dose vs. the higher one.

**Figure 5:**
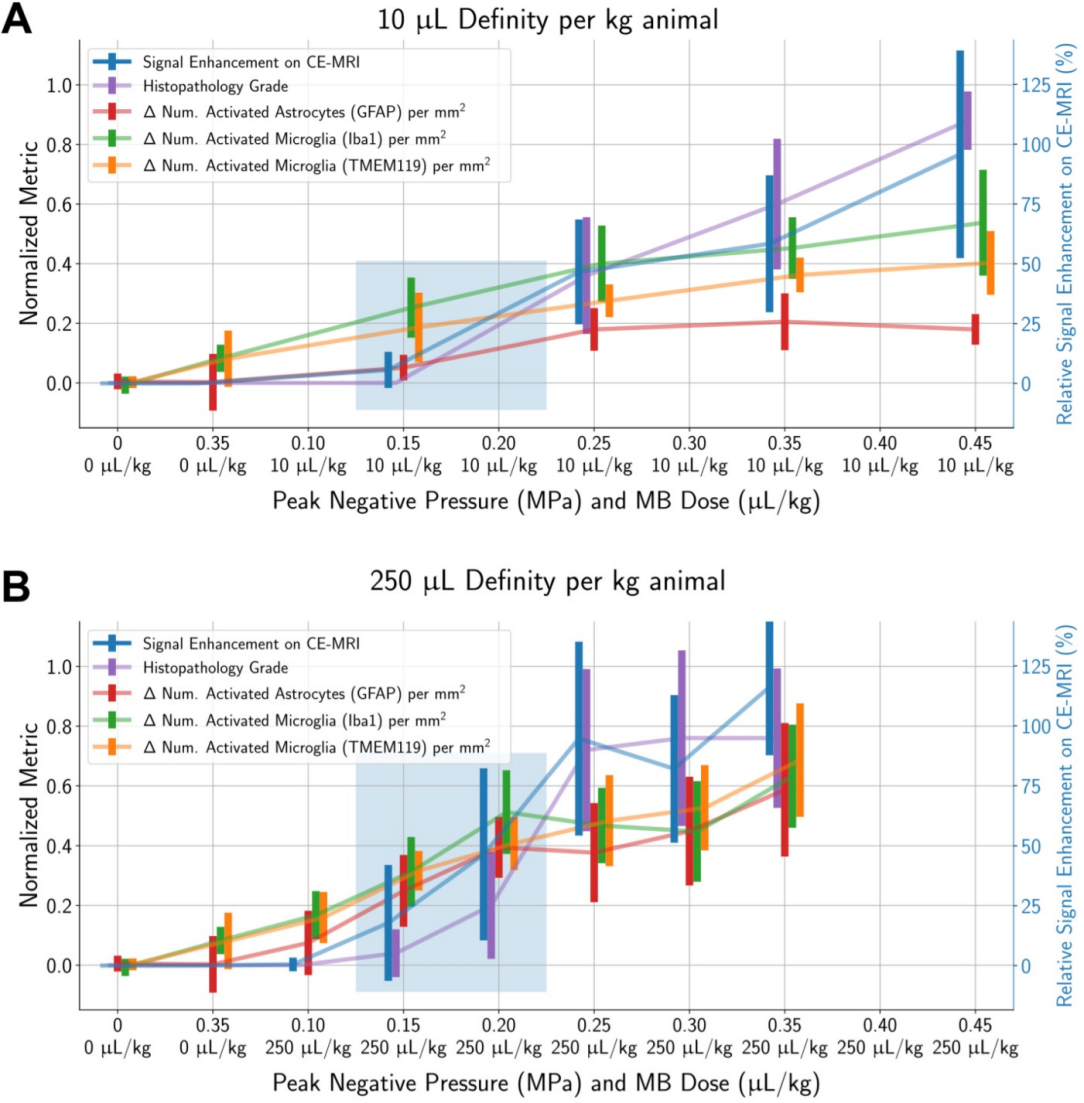
Signal enhancement on CE-MRI, histopathology grade, and the number of activated microglia and astrocytes (in the sonicated hemisphere relative to the control hemisphere) all increase with higher applied PNPs. All metrics (except for signal enhancement on CE-MRI) were normalized by dividing by the global maximum value of that metric (across all ROIs in all animals). Error bars represent standard deviations of each normalized metric. Highlighted regions show treatment parameters that resulted in BBB opening without a significant proportion of animals with histopathological findings: around 0.15 MPa to 0.20 MPa for both MB doses of 10 µL/kg and 250 µL/kg. Finally, there were significantly more activated microglia and astrocytes in the sonicated hemisphere relative to the untreated hemisphere at all PNPs for both MB doses, even in cases when no microhemorrhage was observed. Midlines are situated at the mean normalized metrics for each group, and error bars represent standard deviations.

To determine a suitable PNP range for each MB dose where BBB opening was achieved without significant tissue damage, histopathological grading and differences in activated cell counts between the sonicated vs. untreated hemispheres were normalized by dividing by the global maximum relative signal enhancement (the maximum value across all ROIs in all animals) and plotted alongside relative signal enhancement on CE-MRI (unnormalized) with respect to applied PNP (Figure 5). When using a MB dose of 10 µL/kg, an applied PNP of 0.15 MPa resulted in animals with successful BBB opening without presence of microhemorrhage or edema. Similar findings were observed when using a MB dose of 250 µL/kg; BBB opening was observed with minimal or no histopathological findings in animals treated with an applied PNP of 0.15 MPa. Nonetheless, animals treated with these parameters had significantly more activated microglia and astrocytes in the sonicated hemisphere compared to the untreated one (see summary statistics and *p*-values from Wilcoxon paired-sample tests presented in Table S2).

**Figure 6:**
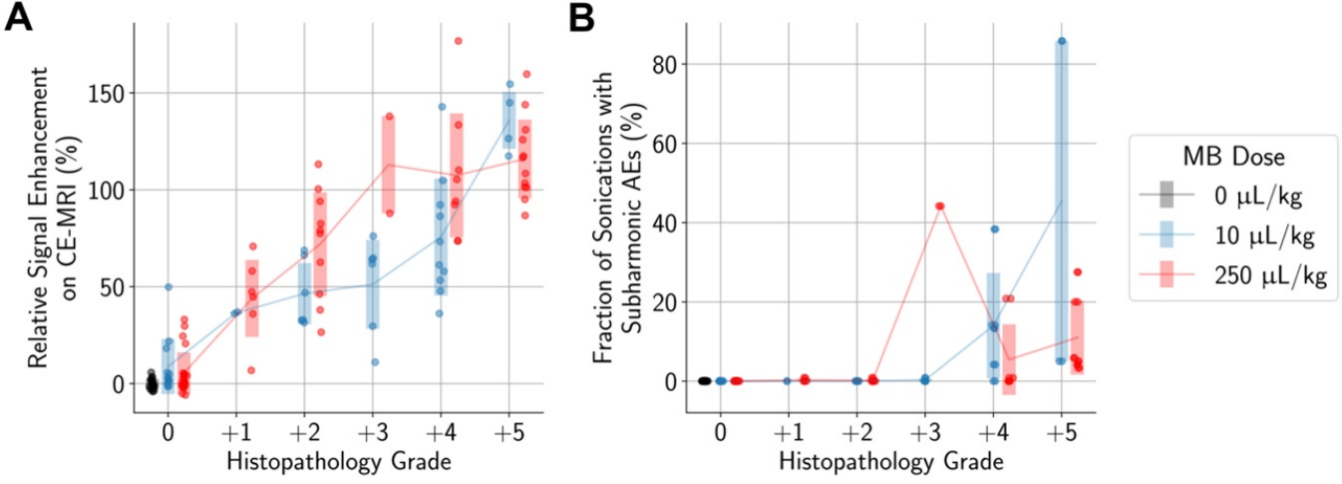
More severe histopathological grading was significantly associated with (A) higher relative signal enhancement on CE-MRI and (B) presence of subharmonic AEs, independent of MB dose. Midlines are situated at the mean for each group, and error bars represent standard deviations.

Unsurprisingly, animals with more severe histopathological grades also had greater relative signal enhancement on CE-MRI, and subharmonic AEs were associated with treatments that resulted in marked (+4) or severe (+5) histopathology grades (Figure 6). Differences in MB dose did not significantly affect these findings; similar CE-MRI signal enhancements were observed between animals with the same histopathology grade, even if they were treated with different MB doses. Subharmonic AEs were also primarily detected at the same histopathology grades of +4 and +5 using both MB doses.

**Figure 7:**
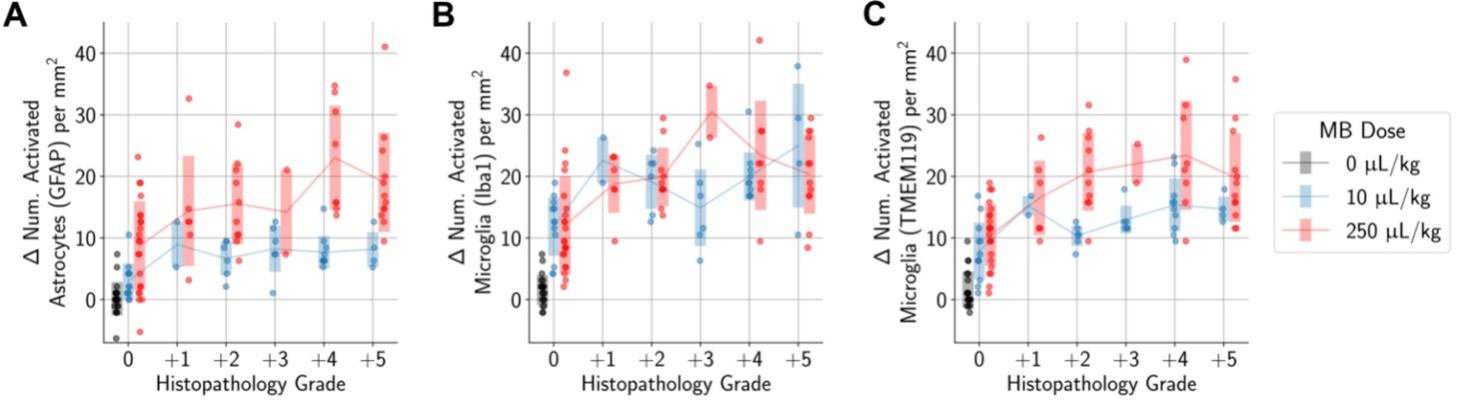
More severe histopathological grading was significantly associated with greater differences in the numbers of activated microglia (A, B) and astrocytes (C) between sonicated and untreated hemispheres. When considering only animals with no histopathological findings, there were still statistically significant differences observed between animals administered MB compared to control animals. Midlines are situated at the mean for each group, and error bars represent standard deviations.

Higher differences in the numbers of activated microglia and astrocytes in the sonicated vs. untreated hemispheres were also significantly associated with histopathology grading (Figure 7). However, there were still increased numbers of activated microglia and astrocytes in the sonicated hemisphere relative to the untreated hemisphere in animals where there were no histopathological findings. These increases were statistically significant when compared to control animals.

### Microglia and astrocyte activation associated with CE-MRI, but not AEs

**Figure 8:**
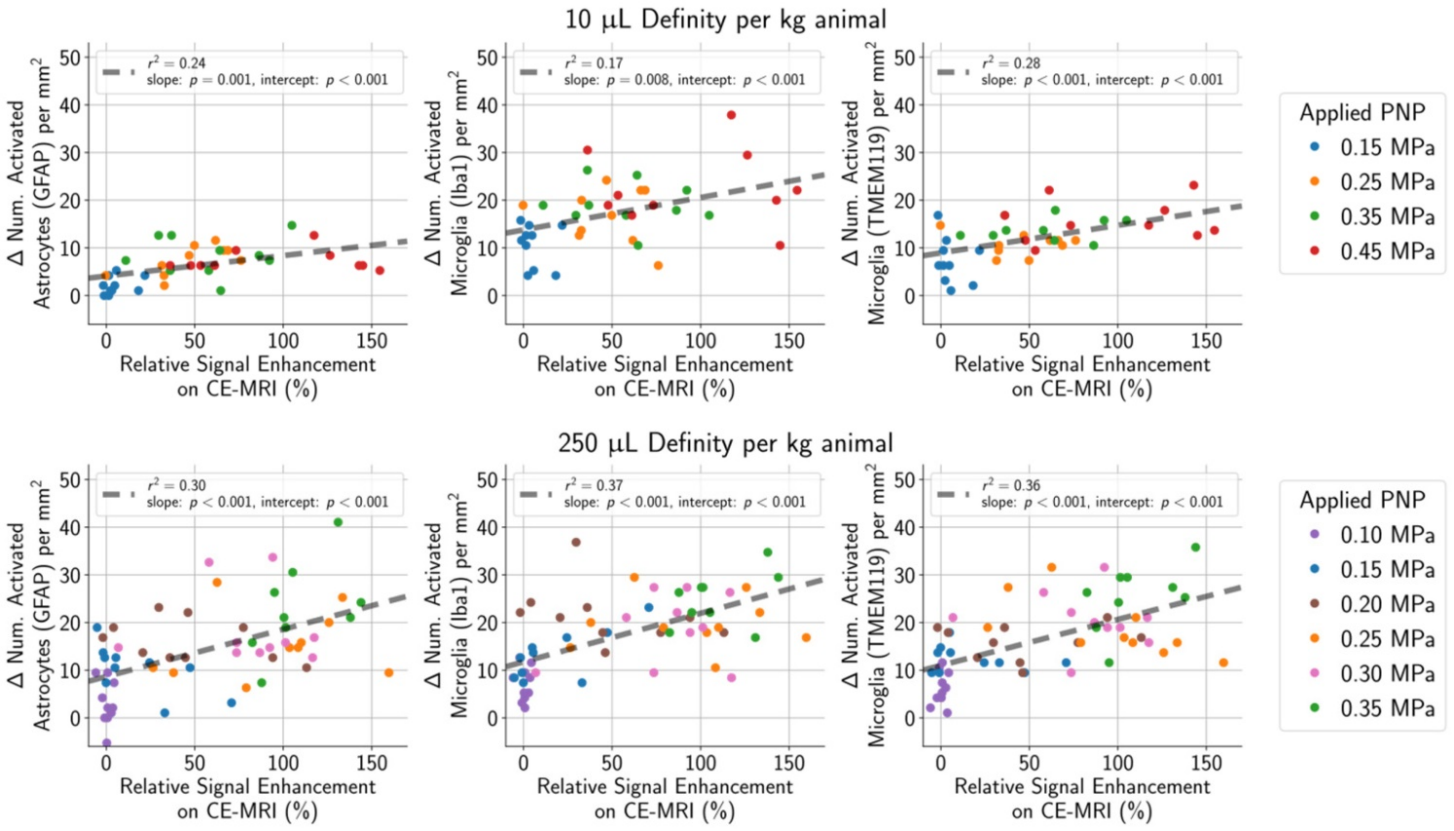
(top row) In animals given a MB dose of 10 µL/kg, the difference between the number of activated microglia and astrocytes per mm^2^ in the sonicated hemisphere relative to the untreated hemisphere was moderately correlated (GFAP stain: r^2^ = 0.24, Iba1 stain: r^2^ = 0.17, TMEM119 stain: r^2^ = 0.28) with the degree of BBB opening (quantified as the amount of relative signal enhancement on CE-MRI). (bottom row) The correlation was slightly stronger in animals given the higher MB dose of 250 µL/kg (GFAP stain: r^2^ = 0.30, Iba1 stain: r^2^ = 0.37, TMEM119 stain: r^2^ = 0.36).

**Figure 9:**
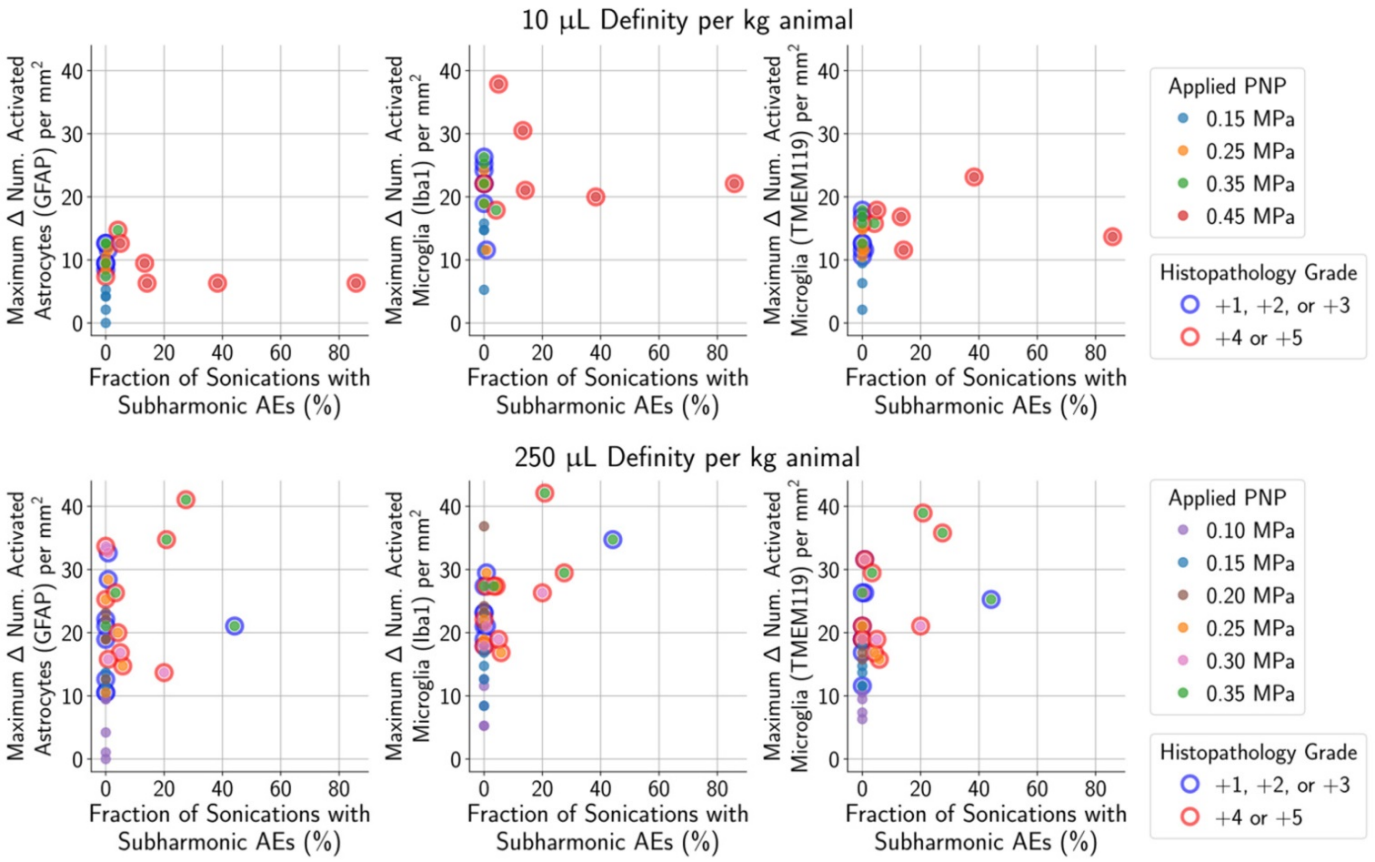
For animals given a MB dose of 10 µL/kg (top row) or 250 µL/kg (bottom row), the difference between the numbers of activated microglia and astrocytes in the sonicated vs. untreated hemispheres were not associated with the presence of subharmonic AEs in animals with histopathological findings ranging from grade 0 (no outline) to grades +1/+2/+3 (blue outline). However, the presence of subharmonic AEs was strongly associated with marked and severe histopathological findings (grades +4/+5, red outline), which were accompanied by greater differences in the number of activated cells.

**Figure 10:**
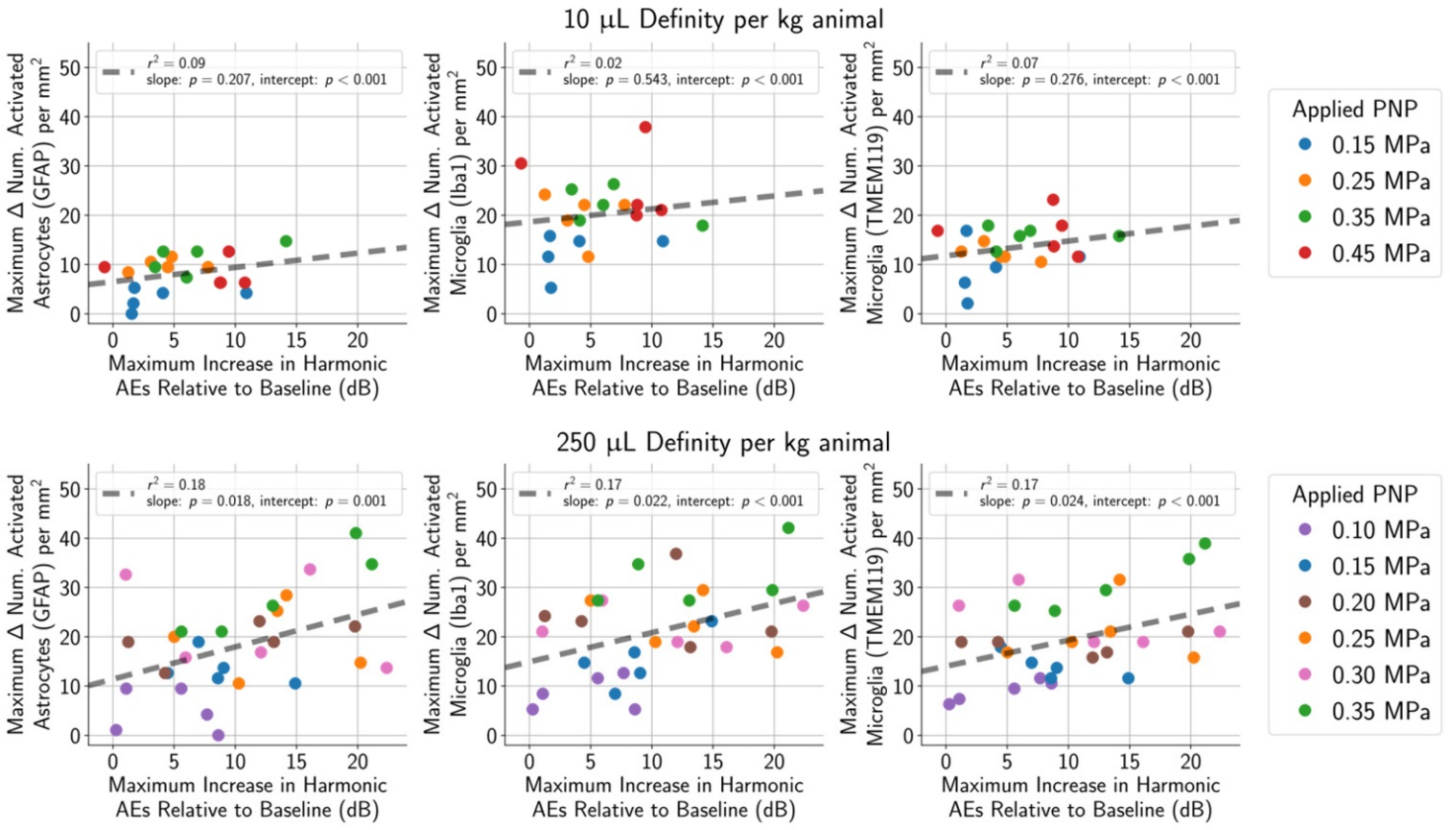
Harmonic AEs were observed to peak approximately 10 s to 40 s following MB bolus injection, and the maximum increase in harmonic AEs (relative to baseline) was recorded for each treatment. (top row) Using a MB dose of 10 μL/kg, the maximum increase in harmonic AEs (relative to baseline) was not associated with the number of activated astrocytes (r^2^ = 0.07 for GFAP) or microglia (r^2^ = 0.09 for Iba1, r^2^ = 0.02 for TMEM119). (bottom row) The association was slightly stronger when using a higher MB dose of 250 μL/kg (r^2^ = 0.18 for GFAP, r^2^ = 0.17 for Iba1, r^2^ = 0.17 for TMEM119).

One goal of the study was to determine if the degree of tissue damage or cellular activation could be predicted using common outcome metrics such as signal enhancement on CE-MRI (Figure 8), subharmonic (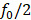) AEs (Figure 9), or harmonic (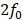) AEs (Figure 10). The degree of BBB opening, as quantified by the signal enhancement on CE-MRI on the sonicated hemisphere relative to the control hemisphere, was moderately associated with the number of activated microglia and astrocytes per mm^2^ on the sonicated vs. untreated hemisphere (Figure 8). As expected, the number of activated cells increased for regions with higher signal enhancement. This was true for both MB dosages, though correlations were stronger when using the higher MB dose.

As mentioned previously, subharmonic AEs were detected in the majority of animals with marked (grade +4) or severe (grade +5) histopathological findings, independent of the MB dose (Figure 9). However, subharmonic AEs were not observed in animals with less severe histopathological grades of +2 or +3, meaning they were not predictive of glial activation when using treatment parameters designed to avoid tissue damage.

Harmonic AEs were observed to peak approximately 10 to 40 s following administration of the MB bolus, and this increase in harmonic AEs relative to baseline was recorded for each treatment. Relative increases in harmonic AEs were not correlated with the number of activated microglia and astrocytes in animals given the lower MB dose of 10 µL/kg (Figure 10, top row), and correlations were only mild in animals given the higher MB dose of 250 µL/kg (Figure 10, bottom row). However, when only considering animals treated using PNPs that did not result in tissue damage, none of these outcome metrics were correlated to glial activation.

## Discussion

Our work has shown that histologically there was an increase in activated microglia and astrocytes in mouse brain tissue one hour following BBB opening, with a significant positive correlation between the applied PNP and the number of activated cells in the sonicated hemisphere relative to the untreated hemisphere (Figure 4). Previous studies have shown that the treatment protocol can significantly impact the degree of potential tissue damage and inflammatory response, with application of a relatively high PNP and high MB dose resulting in significant pathological changes 17. We therefore investigated the effects of a range of PNPs and two MB doses, to produce a variety of treatment outcomes, spanning treatments that failed to open the BBB to treatments with BBB opening alongside significant tissue damage. With this approach, we were able to identify a PNP of 0.15 MPa for each MB dose where BBB opening did not result in significant tissue damage, though activated microglia and astrocytes were still observed.

The goal of our study was to detect acute and occasionally transient morphological activation of microglia and astrocytes one hour following BBB opening. Additional studies are necessary to determine whether minor increases in the number of activated microglia have biological significance, as glial activation can result in both beneficial and detrimental effects depending on the context and duration 37-40. However, while our study only looked at the single time point of one-hour post BBB opening, it will be necessary to examine multiple time points to determine the longevity of the cellular activation and subsequent additional pathologies that may arise (i.e. influx of inflammatory cells). Therefore, we plan to investigate how the inflammatory response changes over time 6- or 24- hours post treatment, similar to previous studies 16,17. This will provide insight as to whether the inflammatory response recovers quickly or persist in response to different treatment parameters.

As *in situ* acoustic pressures cannot be easily predicted in humans due to inter-subject variability of skull structure and composition 22, we investigated whether any outcome metrics, obtained without knowledge of the *in situ* PNP, were predictive of activation of microglia and astrocytes. Relative signal enhancement on CE-MRI was found to be most correlated with glial activation, though these correlations may have been skewed by treating the animals with applied PNPs above the suitable range. Subharmonic AEs were only detected in animals with marked (+4) or severe (+5) histopathology grades, but not in animals with histopathology grades of +2 or +3. This may have been due to the limited sensitivity of the PCD, which had a bandwidth that did not include the subharmonic frequency. Repeating this study with a more sensitive PCD could potentially unveil the presence of subharmonic AEs in animals with mild (+2) or moderate (+3) histopathology grades. However, a more sensitive PCD would not have reduced the strong association between marked (+4) or severe (+5) histopathology grades that was observed with the less sensitive PCD used in this study. Future studies will improve on the PCD's sensitivity to these subharmonic frequencies, which could enable better prediction of the degree of glial activation and potentially allow us to detect weaker AEs associated with mild or moderate histopathology grades.

This study applied a constant acoustic pressure for all sonications within a single treatment, and cavitation feedback monitoring was not used to control for the applied acoustic pressure. In proposed open-loop feedback-controlled BBB opening protocols, the applied acoustic pressure is linearly ramped up until subharmonic 41 or ultraharmonic 10 AEs are detected, then half of the maximum acoustic pressure is applied for the remainder of the treatment. It has been shown that treatments using this open-loop feedback system in combination with a low MB dose results in safer BBB opening compared to applying a constant PNP of 0.30 MPa following administration of a higher MB dose 17, where the average applied PNP using the feedback system was around 0.20 MPa. However, it is unclear if there is a significant difference between treatments implementing PCD-based feedback systems and treatments using a constant PNP yet applying the same average PNP of 0.20 MPa. In this study, a range of constant PNPs were applied, and BBB opening without hemorrhage or edema was still achieved using an applied PNP of 0.15 MPa with a MB dose of 10 µL/kg. Histopathological findings, such as edema, also appear to be more likely when higher MB doses are used, even when using PCD-based feedback 17. This also agrees well with what was observed in our study, where microhemorrhage and edema was observed in a subset of animals given a MB dose of 250 µL/kg but not animals given a MB dose of 10 µL/kg when using an applied PNP of 0.15 MPa.

Finally, there are several different implementations of PCD-based feedback control, including systems that are closed-loop 42. Different systems look at various portions of the acoustic spectrum, including subharmonics, harmonics, and ultraharmonics. All systems have demonstrated the ability to achieve BBB opening without hemorrhage or edema, but it is unknown if there is a difference in the corresponding inflammatory response. The degree of activation of microglia and astrocytes could be primarily determined by the degree of BBB opening, and not the specific sonication parameters used. It could be worthwhile to investigate whether there are differences in the inflammatory response depending on the type of feedback system used.

A limitation of this study was that treatments were susceptible to standing wave artifacts due to the low excitation frequency used (650 kHz), the relatively long pulse length (10 ms), and the small size of the mouse brain 43. The presence of standing waves occurs due to reflections at the boundary between the skull and the brain, resulting in higher *in situ* peak acoustic pressures than what was applied. Bright bands, separated by approximately half a wavelength, were observed on CE-MRI in a few animals, which are strongly indicative of standing wave artifacts. These in turn contributed to uncertainty in the exact acoustic pressure delivered into the brain. We observed consistent microhemorrhages on histology at applied PNPs of 0.25 MPa and higher, but other studies that were not susceptible to standing wave artifacts might observe hemorrhage only at higher applied PNPs. However, the goal of this study was to investigate the degree of activation of microglia and astrocytes for a wide range of treatment outcomes and the relationship with the applied acoustic pressure, but not necessarily dependent on the exact value of the applied PNP. Significant differences in the number of activated microglia and astrocytes in the sonicated hemisphere relative to the control hemisphere were found even in animals sonicated at 0.15 MPa, which was not high enough to cause microhemorrhages at a MB dose of 10 µL/kg (Figure 5). We also found a slight association between the number of activated cells and metrics that are independent of applied PNP, such as signal enhancement on CE-MRI (Figure 8). Thus, the presence of these standing wave artifacts does not discount the fact that there was activation of microglia and astrocytes associated with BBB opening, and that it increased with applied acoustic pressure with both microbubble doses.

Another limitation was that histological analyses were performed based on a single brain slice per animal. Though the diameter of the focal spot (2.7 mm at full-width half-maximum) was large relative to the targeted region of the mouse brain, the analyzed slice may not always have been at the center of the focal spot, resulting in underestimation of the severity of histopathological grading and the degree of cellular activation. This would have disproportionately affected animals treated with either low applied PNPs or without MB administration, as there was no signal enhancement on CE-MRI to help guide tissue slicing due to the intact BBB. Finally, the approach used to assess microglial and astrocyte activation was limited, as assessments were based on morphological evaluation of a small number of cells per animal. Histological analyses were performed on a single brain slice per animal, which likely resulted in an underestimation of the number of activated microglia and astrocytes compared to those made using stereological techniques 44. Future studies will include proteomic and transcriptomic analyses to provide a more comprehensive understanding of the activation of the inflammatory response following ultrasound-mediated BBB disruption with varying treatment parameters.

Microglia and astrocytes coordinate their functions to maintain homeostasis in the central nervous system, and they respond in synchrony to acute and chronic injury by direct signaling through cytokines and other molecules. As we search for less invasive treatments to transport large therapeutic molecules into the brain, it is necessary to understand the behavior of these cells in health and disease. Currently, relatively little is known regarding how microglia and astrocytes act following pFUS-mediated blood-brain barrier opening. This study provides further insight into the relationship of glial cell activation with other experimental parameters, such as applied PNP and MB dose, providing additional information toward the goal of delivering safe treatments into the brain.

## Conclusions

Herein, we identified a suitable PNP range from 0.15 MPa to 0.20 MPa where BBB opening was observed with minimal histopathological lesions. Using this PNP with a MB dose of 10 µL/kg, minimal activation of microglia and astrocytes were also observed one hour following BBB opening. Using a higher MB dose of 250 µL/kg, the same applied PNPs produced similar results in signal enhancement on CE-MRI, activation of microglia and astrocytes, and histopathological grading. Higher applied PNPs increased the degree of all of these findings, with greater increases when using the higher MB dose.

The number of sonications with subharmonic AEs was not predictive of the number of activated microglia or astrocytes in the safe PNP range (histopathology grades of 0, +1, or +2) of 0.15 MPa. The maximum increase in harmonic AEs (relative to baseline) was also not a reliable predictor of the number of activated microglia or astrocytes. Similarly, enhancement on CE-MRI was not predictive of the number of activated microglia and astrocytes within the safe PNP range. The presence of subharmonic AEs was strongly associated with marked (grade +4) and severe (grade +5) histopathological grades, independent of MB dose. Future work will assess the biological and behavioral relevance of these findings.

## Supplementary Material

Supplementary figures and tables.

## Figures and Tables

**Table 1:** Criteria used for semiquantitative grading of histopathological lesions. Observed lesions consisted of differing levels of microhemorrhage and edema. No lesions were observed in the parenchyma of the untreated, contralateral hemisphere of the brain.

Histological Grade	Grading Criteria
0 (None)	No changes observed in the brain parenchyma
+1 (Minimal)	Rare/scattered (three or fewer) microhemorrhages accompanied by minimal edema
+2 (Mild)	Multiple (up to ten) microhemorrhages accompanied by mild edema
+3 (Moderate)	Multiple (up to ten) microhemorrhages accompanied by moderate edema
+4 (Marked)	More than ten microhemorrhages accompanied by marked edema
+5 (Severe)	More than ten microhemorrhages accompanied by severe, regionally extensive edema

## Abbreviations

AE: acoustic emission
BBB: blood-brain barrier
CE-MRI: contrast-enhanced magnetic resonance imaging
GFAP: glial fibrillary acidic protein
Iba1: ionized calcium binding adaptor molecule 1
MB: microbubble
pFUS: pulsed focused ultrasound
PNP: peak negative pressure
TMEM119: transmembrane protein 119
