# Supplementary material for: Histologic evaluation of activation of acute inflammatory response in a mouse model following ultrasound-mediated blood-brain barrier using different acoustic pressures and microbubble doses
Source: Nanotheranostics. 2020 Jul 14;4(4):210–23. doi: 10.7150/ntno.49898 (PMC7425053; doi:10.7150/ntno.49898)
Supplement: Supplementary file 1 — Supplementary figures and tables. [file ntnov04p0210s1.pdf]

Supplementary Figures and Tables

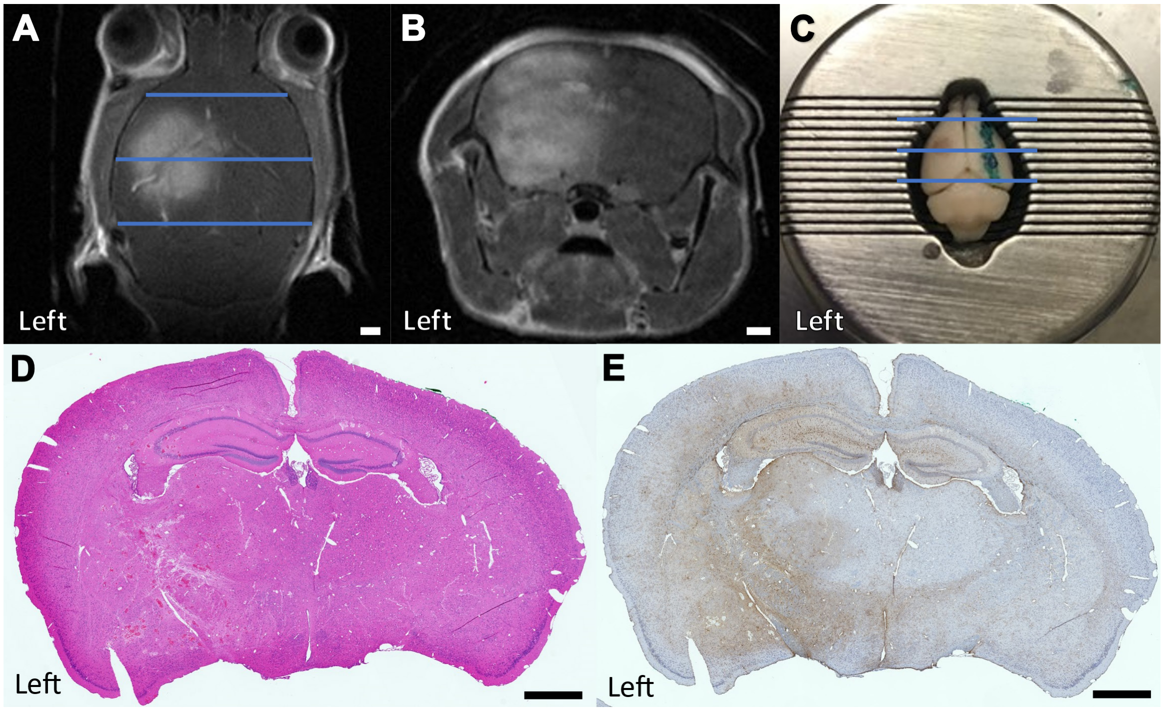

Figure S1. (A) Protocol used to obtain brain slices for histology. MR images were flipped from radiological convention such that the left hemisphere corresponded to the left of the image. Axial (A) and coronal (B) CE-MRI slices were used alongside hemorrhage visible on the surface of the brain (C), if present, to help guide the slicing process. Following perfusion, the harvested brain (with green dye applied to identify the untreated hemisphere) was positioned in a brain slicing mold (C), with blue lines denoting where cuts were made to yield 3 mm thick coronal slices. Brain slices were routinely processed for H&E and IHC (D, E). All scalebars represent 1 mm.

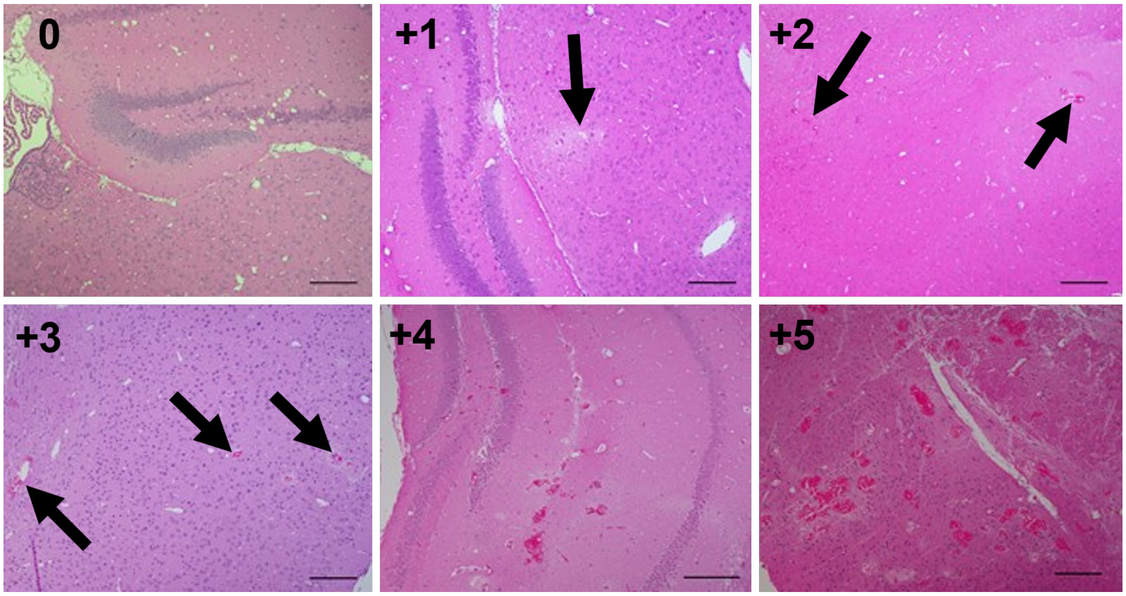

Figure S2. Representative H&E-stained histology images (10× magnification) for each histopathological grade: 0 (none), +1 (minimal), +2 (mild), +3 (moderate), +4 (marked), and +5 (severe). Scalebar = 100 µm.

Table S1. Percentage of mice with BBB opening on MRI and the average histopathology grade observed at applied PNP. Signal enhancement on CE-MRI resulting from BBB opening was observed with both doses of MB at a PNP of 0.15 MPa. Changes in the parenchyma were always more severe at high doses of MB al the same PNP. No pathological findings were observed in the control (non-sonicated) contralateral hemispheres.

PNP (MPa)	MB dose (µL/kg)	Proportion of animals with BBB opening on CE-MRI	Average histopathology grade
0	0 (saline)	0/5	0
0.35	0 (saline)	0/5	0
0.15	10	2/5	0
0.25	10	5/5	1.8
0.35	10	5/5	3
0.45	10	5/5	4.4
0.10	250	0/5	0
0.15	250	3/5	0.2
0.20	250	4/5	1
0.25	250	5/5	3.6
0.30	250	5/5	3.8
0.35	250	5/5	3.8

Table S2. Summary statistics of the number of activated microglia and astrocytes per mm^2 observed in each 1.32 mm by 0.72 mm field of view. Means and standard deviations were computed across all brain regions (two regions in each hemisphere per animal) for each treatment group.

PNP (MPa)	MB dose (µL/kg)	Number of activated microglia (Iba1+) per mm^2			Number of activated microglia (TMEM119+) per mm^2			Number of activated astrocytes (GFAP+) per mm^2		
		Control hemisphere	Sonicated hemisphere	*p*-value	Control hemisphere	Sonicated hemisphere	*p*-value	Control hemisphere	Sonicated hemisphere	*p*-value
0	0 (saline)	18.31 ± 4.30	17.99 ± 3.66	0.376	9.36 ± 2.19	9.47 ± 2.22	0.639	6.21 ± 1.26	6.42 ± 1.35	0.441
0.35	0 (saline)	34.72 ± 4.32	38.19 ± 5.04	0.795	32.93 ± 3.58	36.09 ± 4.89	0.005	12.52 ± 6.40	12.63 ± 4.94	0.032
0.15	10	17.36 ± 4.01	27.99 ± 7.09	0.007	10.21 ± 4.76	17.47 ± 8.24	0.005	6.84 ± 2.29	8.94 ± 2.68	0.005
0.25	10	22.20 ± 5.28	39.04 ± 8.48	0.005	13.89 ± 5.67	24.62 ± 6.86	0.005	6.84 ± 1.51	14.20 ± 3.30	0.005
0.35	10	24.31 ± 4.61	43.35 ± 5.15	0.005	13.68 ± 2.53	27.78 ± 3.82	0.005	6.84 ± 2.99	15.26 ± 5.60	0.005
0.45	10	22.10 ± 4.78	44.72 ± 8.74	0.005	12.73 ± 2.50	28.41 ± 6.16	0.005	7.58 ± 4.17	14.94 ± 4.70	0.005
0.1	250	30.41 ± 5.42	37.46 ± 6.20	0.049	24.20 ± 5.15	30.41 ± 6.48	0.005	20.83 ± 5.99	23.88 ± 7.41	0.005
0.15	250	36.41 ± 10.05	49.56 ± 10.71	0.005	21.89 ± 4.75	34.20 ± 4.61	0.005	13.47 ± 4.08	23.67 ± 6.49	0.005
0.2	250	30.62 ± 7.83	52.19 ± 8.42	0.005	19.89 ± 3.76	35.77 ± 7.08	0.005	16.10 ± 4.33	32.30 ± 7.36	0.005
0.25	250	26.41 ± 4.56	46.09 ± 9.12	0.005	17.36 ± 2.23	36.20 ± 6.26	0.005	12.73 ± 4.39	28.20 ± 7.76	0.005
0.3	250	32.30 ± 8.78	51.14 ± 12.68	0.005	19.89 ± 3.08	40.40 ± 4.84	0.005	10.31 ± 3.96	28.72 ± 7.70	0.005
0.35	250	29.04 ± 12.02	55.66 ± 15.37	0.005	16.41 ± 5.25	43.14 ± 10.40	0.005	10.94 ± 6.60	35.04 ± 10.87	0.005
